# Bone marrow‐derived mesenchymal stem cells attenuate complete Freund's adjuvant‐induced inflammatory pain by inhibiting the expression of P2X3

**DOI:** 10.1111/cpr.13461

**Published:** 2023-03-27

**Authors:** Yifan Deng, Dongdong Yuan, Zhizhao Deng, Jianfen Liang, Zhenye Zhang, Ziqing Hei, Xiang Li

**Affiliations:** ^1^ Department of Anesthesiology the Third Affiliated Hospital of Sun Yat‐Sen University Guangzhou 510630 China

## Abstract

Bone marrow‐derived mesenchymal stem cells (BMSCs) show a good property for pain treatment by modulating inflammatory response. However, the underlying therapeutic effect and related mechanism of BMSCs on inflammatory pain remain unclear. Therefore, we explored the function and potential mechanism of BMSCs performing in a complete Freund's adjuvant (CFA)‐induced inflammatory pain model in this study. Here, BMSCs were injected into the CFA‐treated rats, and we used behavioural tests to evaluate the changes in hypersensitivity. High‐throughput sequencing was used to screen out the hub genes. Molecular biology experiments were performed to detect the level of P2X3 or inflammatory mediators in rats and observed the distribution of P2X3 in neural cells. Furthermore, the function of the P2X3 was explored via inhibitor and activator experiments. Finally, we found that BMSCs alleviated hyperalgesia and spinal levels of pro‐inflammatory factors in CFA‐treated rats. High‐throughput sequencing showed that P2X3 and P2X7 were identified as hub genes, and only the expression level of P2X3 was significantly down‐regulated after BMSCs treatment. Immunohistochemistry showed that P2X3 mainly colocalized with microglia and astrocytes. The levels of P2X3 and pro‐inflammatory factors were all significantly reduced after BMSC injection. Moreover, similar attenuation was found in the CFA‐treated rats after injecting the P2X3 inhibitor, and a P2X3 antagonist reversed the attenuation induced by the BMSCs. These findings suggest that BMSCs exerted a therapeutic effect on inflammatory pain by inhibiting the expression of P2X3 and the excessive production of inflammatory mediators was associated with an increased P2X3 level and BMSC therapy reverse these effects.

## Graphical abstract

Bone marrow‐derived mesenchymal stem cells alleviated complete Freund's adjuvant‐induced inflammatory pain by decreasing the levels of P2X3 in the spinal cord of the rats.

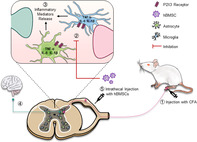

## 1 INTRODUCTION

Inflammatory pain is the most common type of chronic pain seen in the clinic. Inflammation is defined as a primary defence and immune response, and it can result in including pain and swelling.^1^, ^2^ For patients, the most unbearable pain is caused by the allodynia. The allodynia is induced by a chemical, mechanical or thermal stimulus.^3^, ^4^ Over time, allodynia can affect the sleep and cognitive processes of the patients, and mood disorders such as depression and anxiety are frequently diagnosed in these patients.^5^, ^6^, ^7^ Thus, we should attach great importance to this disease.

The traditional treatment for inflammatory pain is medical therapy, including opioids and nonsteroidal anti‐inflammatory drugs (NSAIDS).^8^, ^9^ Although many medicines can be used to alleviate inflammatory pain, they fail to provide sustained relief in patients. Moreover, their side effects needed to be considered; for example, opioids repress the immune response, and NSAIDS are often accompanied by ulcer formation.^10^, ^11^ Therefore, new therapies need to be explored to treat inflammatory pain.

Mesenchymal stem cells (MSCs) can be extracted from numerous mesodermal tissues, including dental pulp, placenta, and bone marrow.^12^ MSCs can preserve regenerative function after cryopreservation, improving synaptic transmission and repairing neuronal networks.^13^, ^14^ In recent years, numerous reports have shown that MSCs attenuated hypersensitivity induced by neuropathic pain.^15^, ^16^, ^17^, ^18^ In addition to neuropathic pain that is commonly resulted from a lesion in the central or peripheral branches of nerves, inflammatory pain is characterized by high levels of inflammatory components, which are increased by activation of the immune system and/or by tissue injury or infection.^19^, ^20^ From this perspective, understanding the effects of BMSCs on inflammatory pain may help expand the knowledge of BMSCs in the field of analgesia.

Prior evidence has demonstrated that MSCs can attenuate mechanical and thermal hypersensitivity by hindering the expression of the purinergic receptors P2X4 and P2X7.^21^ Moreover, Inoue et al. reported that P2X4 or P2X7 played a critical role in transmitting nociceptive signals in neuropathic pain.^22^ In inflammatory pain, P2X3, P2X4, and P2X7 have been shown to be the main transitional signal receptor mediating the pathway activation.^23^, ^24^, ^25^ An inflammatory pain model can be induced by a complete Freund's adjuvant (CFA) reagent, and these models are among the best for studying chronic inflammation.^26^, ^27^ In summary, we postulated that MSCs alleviated the mechanical and thermal hypersensitivity induced by inflammatory pain by reducing the levels of P2XR.

In the present study, we chose human bone marrow‐derived mesenchymal stem cells (hBMSCs) as the experimental treatment, because of their easy accessibility, multipotency, and low immunogenicity.^28^ Besides, we utilized high‐throughput sequencing to screen out the hub genes that change differentially in the spinal cord before/after injecting hMSCs in inflammatory pain rats. Our study aimed to determine the effect of hBMSCs on inflammatory pain and identify which P2XR participates in this therapeutic effect.

## 2 MATERIALS AND METHODS

### 2.1 Animal preparation

Six‐week‐old male Sprague–Dawley rats (200 ± 20 g) were purchased from SPF (Beijing) Biotechnology Co., Ltd (production licence number: SCXK [Jing] 2019‐0010, Beijing, China; Wilmington, MA). All the rats were housed individually in a cage in the Animal Experimental Center of South China Agricultural University in a 12‐h light/dark cycle with a suitable temperature of 23–25°C and free access to food and water. All animals received human care and all protocols in this study conformed to the ethics guidelines of the 1975 Declaration of Helsinki and were approved by the Experimental Animal's Ethics Committee of the South China Agricultural University (No. 2022d055) in May 2022. Animal welfare‐related assessments, measurements and interventions (e.g., humane endpoints) are in compliance with the ARRIVE guidelines.^29^

### 2.2 Experimental design, the establishment of the inflammatory pain model and drug treatment

Using a random number table generated by SPSS version 25.0 software, 96 rats were randomly divided into eight groups (*n* = 12 per group) as follows: sham group, CFA group, CFA+Veh‐1 (William's E Medium) group, CFA+hBMSCs group, CFA+A317491 group, CFA+Veh‐2 (saline) group, CFA+ hBMSCs+Veh‐2 group, CFA+hBMSCs+α β‐me ATP group. Moreover, additional 10 rats were randomly divided into CFA (*n* = 5) or CFA+hBMSCs (*n* = 5) groups to carry out mRNA sequencing.

**FIGURE 1:**
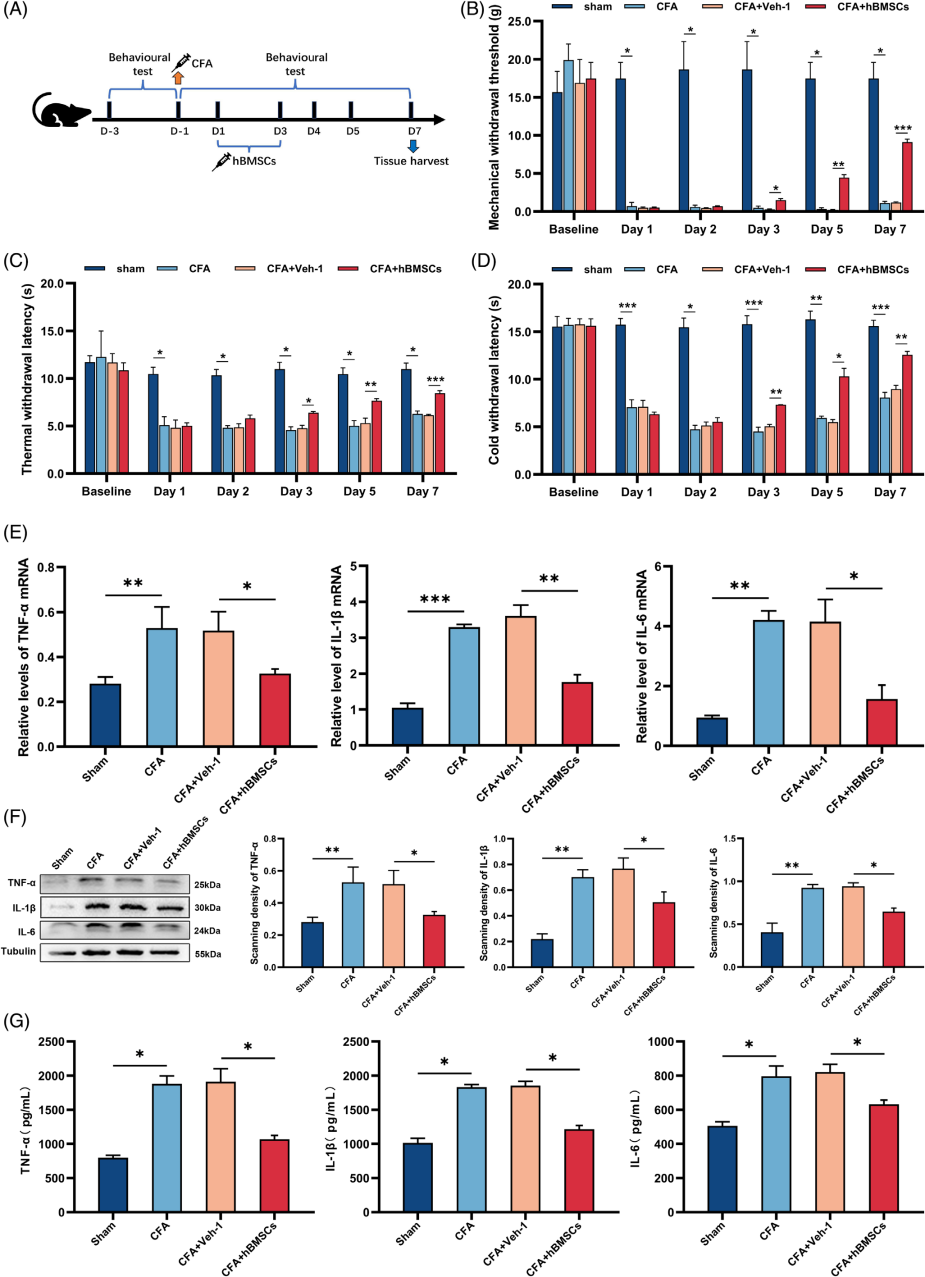
hBMSCs alleviated hyperalgesia and inflammatory cytokine increase induced by CFA. (A) The procedure of intrathecally administrating hBMSCs. (B–D) Behavioural tests showed the analgesic effect of hBMSCs on the mechanical, thermal, and cold withdrawal thresholds of inflammatory pain. The CFA+hBMSCs group exhibited a significant increase in mechanical, thermal, and cold withdrawal thresholds compared with those of the CFA and CFA+Veh‐1 groups. (E) The qRT–PCR results in the gene expression of proinflammatory cytokines. The mRNA levels of IL‐1β, TNF‐α, and IL‐6 were increased in the CFA and CFA+ Veh‐1 groups, while they were decreased in the CFA+hBMSCs group. (F) Representative western blot image showing the inflammatory cytokines and quantification of their relative expression in each group. Tubulin was the loading control. Changes in the levels of each inflammatory cytokine were the same as those obtained by qRT–PCR. (G) The levels of inflammatory cytokines were determined by ELISAs. The changes in the levels of each inflammatory cytokine were the same as they were in the western blot assay. *p < 0.05, **p < 0.01, ***p < 0.001. *n* = 3 each group.

A persistent inflammatory pain model was established according to a previously reported procedure.^30^ Briefly, 0.1 mL CFA (Sigma–Aldrich, St. Louis, MO) was injected into the plantar surface of the left hind paw of rats under anaesthesia with 1.5–2.5% isoflurane treatment. For the sham group, an equal volume of saline was injected in the same place of the left hind paw on the rats as the injection of CFA with or without treatment. Then, behavioural tests including mechanical sensitivity measurements and thermal sensitivity measurements were performed once every day to evaluate the model. Rats were intrathecally injected at the L3‐4 or L4‐5 spinal level with 1 × 10^6^/10 μL hBMSCs once per day consecutively for three consecutive days after CFA administration, or injected with 300 nmol A317491 (the specific P2X3 antagonist, Sigma–Aldrich, Saint Louis, MO) once on Day 3 after CFA administration or 100 nmol α β‐me ATP (the specific P2X3 agonist, Sigma–Aldrich) once on day 3 after CFA administration. A317491 and α β‐me ATP were diluted with sterile 0.9% saline (Figure 1A). The rat tail‐flick reflex is considered to be the sign that injected substance entered the subarachnoid space. The rats were sacrificed by an intraperitoneal overdose (1.5 mL) of 3% pentobarbital sodium, and the L4‐5 levels of lumbar enlargement were harvested for further studies on Day 7.

### 2.3 hBMSC culture and preparation

The isolated hBMSCs were purchased from Cyagen Biosciences (Batch number 211102H61, Guangzhou, China), and the test results of surface markers and differentiation capacity of the hBMSCs were provided as the Data S1. The cells were cultured with a MesenCultTM‐ACF Plus Culture Kit (STEMCELL, Vancouver, Canada) supplemented with 5 μg/μL Plasmocin™ Prophylactic (InvivoGen) and in a sterile cell incubator (Thermo Fisher Scientific, Waltham, MA) maintained at 37°C, with 90% humidity and 5% CO_2_. Upon reaching 90% confluence, cells between passage 3 and passage 8 were harvested by ACCUTASE™ (STEMCELL) and suspended in William's E Medium (ThermoFisher) for intrathecal injection. An equal volume of William's E Medium was used as the vehicle.

### 2.4 Behavioural tests

Measurements of mechanical hypersensitivity, thermal allodynia, and cold hyperalgesia were performed to evaluate the sensitivity of rats to pain, as described in a previous study.^31^ To measure the mechanical hypersensitivity, von Frey filaments (North Coast Medical, California) were used to test the sensitivity of the left mid‐plantar surface of the hind paw with forces ranging from 0.16 to 26.0 g. Rats were placed in a tactile test cage for 0.5 h in a room with the temperature maintained at 24°C and with 80% humidity before the test. The moment when the rat was licking its hair or moving was not measured. The reaction of a rat pulling back its left paw before the fibre lasted 5 s was considered a positive result and the corresponding force was recorded. Three consecutive events were recorded at 5‐min intervals, and the mean values were used for further analysis.

Thermal allodynia was measured with a radiant heat apparatus (San Diego University, CA) at a temperature of 50°C. The reaction of a jump or a lick in the left paw was considered to be a positive response. A cut‐off of 20 s was set to prevent cutaneous damage during a negative response. The trial was repeated three times at 15‐min intervals, and the mean values were used for further analysis.

Cold hyperalgesia was measured with a cold plate apparatus (Ugo Basile, Comerio, Italy) at a temperature of 4°C. A reaction of a jump or a lick in the left paw was considered to be a positive response. A cut‐off of 30 s was set to prevent cutaneous damage during a negative response. The trial was repeated three times at 15‐min intervals, and their mean values were used for further analysis.

### 2.5 RNA isolation and sequencing

The spinal samples of the lumbar enlargement of rats were treated with Trizol Reagent (Invitrogen, California, USA) to extract RNA. Using TruSeq® RNA Sample Preparation Kit (Illumina, USA) to create the paired‐end libraries. The poly‐A‐containing mRNA molecules were purified using poly‐T oligo‐attached magnetic beads. Purified libraries were quantified by Qubit® 2.0Fluorometer (Life Technologies, USA) and verified by Agilent 2100 bioanalyzer (Agilent Technologies, USA) to determine the insert size and calculate the mole concentration. Cluster was generated by cBot with the library diluted to 10 pM and then were sequenced on the Illumina HiSeq X‐ten (Illumina, USA).

The concentration of all RNA was determined by NanoDrop 1000 spectrophotometer (ThermoFisher Scientific) and conducted sequencing by Illumina. After that, mRNA samples was purified with the NEBNext Poly(A) mRNA Magnetic Isolation Module (NEB, E7490S), and using Oligo beads to enrich the mRNA. The KAPA Stranded RNA‐Seq Library Prep Kit (Illumina, USA) was used to create the RNA sequence, according to the instruction of the manufacturer. After that, Agilent 2100 Bioanalyzer (Agilent, Germany) was used for control inspection of the library. To create a single‐strand DNA, the DNA fragments in these libraries were treated with 0.1 mol/L NaOH and loaded onto the channels of Illumina flow cell at a concentration of 8 pmol/L, amplified in situ using TruSeq SR Cluster Kit v3‐cBot‐HS (#GD‐401‐3001, Illumina). Finally, these RNAs were sequenced in the Illumina HiSeq 4000 for 150 cycles.

### 2.6 Bioinformatics analysis

The R package DESeq was used to analyse the high‐throughput sequencing data to determine the differentially expressed genes (DEGs) related to BMSCs treatment with the thresholds of |log2FoldChange| > 2 and *p* <0.05.^32^ After that, the Gene ontology (GO) items including biological process (BP), molecular function (MF) as well as cellular component (CC) terms and Kyoto Encyclopedia of Genes and Genomes (KEGG) pathways annotations were enriched for DEGs by clusterProfiler package.^33^ Finally, a protein–protein interaction (PPI) network of the DEGs was established using the STRING database (http://string-db.org/), and the hub genes were regarded as those with a higher connectivity degree in the PPI network.

### 2.7 Quantitative real‐time polymerase chain reaction

The spinal cord samples were homogenized, and total RNA was extracted using a RNeasy Mini Kit (Qiagen GmbH, Hilden, Germany) according to the instructions of the manufacturer. cDNA synthesis was performed using PrimeScript RT Master Mix (Takara Bio, Beijing, China), and real‐time RT**–**PCR was performed on a LightCycler 480 real‐time PCR system (Roche, Rotkreuz, Switzerland) using iTaq Universal SYBR Green Supermix (Bio‐Rad, Hercules, CA, USA). Tubulin was used as the internal control. Primer 5.0 software was used to design the primer sequences, which are listed in Table 1.

**TABLE 1:** Sequences of the primers used for real‐time RT‐PCR.

mRNA	Primers	Sequences (5′‐3′)
P2X3	Upstream	CAGGGCACCTCTGTCTTTGTC
	Downstream	TCAGACACACAGCGGTACT
P2X7	Upstream	ATATCCACTTCCCCGGCCAC
	Downstream	TCGGCAGTGATGGGACCAG
IL‐1β	Upstream	GAAATGCCACCTTTTGACAGTG
	Downstream	TGGATGCTCTCATCAGGACAG
IL‐6	Upstream	AAGAGACTTCCAGCCAGTTG
	Downstream	TGGATGGTCTTGGTCCTTAG
TNF‐α	Upstream	TAGCCCACGTCGTAGCAAAC
	Downstream	ACAAGGTACAACCCATCGGC
Tubulin	Upstream	GCTGTCAACATGGTGCCCTTCC
	Downstream	ATCCTCTTCCTCTTCTGCGGTGGC

### 2.8 Western blot

All tissues taken from the rats were lysed with RIPA buffer and centrifuged for 12 min at 12500*g* to extract the protein. A BCA assay kit (Bio‐Rad, Hemel Hempstead, Herts, UK) was used to determine the concentration of the protein. The protein was run on a 12.5% polyacrylamide gel and subsequently transferred to a 0.45 μm polyvinylidene fluoride membrane (Millipore, Suzhou, China). 5% skim milk diluted with TBST was used to block the membrane for 1 h, and then, the membrane was incubated with primary antibody at 4°C overnight. The following antibodies were used: rabbit anti‐ interleukin (IL)‐1β (1:1000, ABclonal, Hubei, China), rabbit anti‐IL‐6 (1:1000, ABclonal), rabbit anti‐TNF (tumour necrosis factor)‐α (1:1000, ABclonal), rabbit anti‐P2X3 (1:2000, ABclonal), rabbit anti‐P2X7 (1:2000, ABclonal), rabbit anti‐GAPDH (1:5000, ABclonal) and rabbit anti‐Tubulin (1:5000, ABclonal) antibodies. After then, the membranes were incubated with goat anti‐rabbit antibody (1:20000, Invitrogen) for 1 h at room temperature. Images were captured with a Tanon 5500 imaging system (Tanon, Shanghai, China) and band intensities were analysed via the ImageJ software (National Institutes of Health, Bethesda, USA).

### 2.9 Enzyme‐linked immunosorbent assay

Tissues harvested from rats were homogenized in PBS and centrifuged at 4°C for 15 min at 13000*g*. The collected supernatants were used to determine the concentrations of IL‐1β, IL‐ 6, and TNF‐ α with enzyme‐linked immunosorbent assay (ELISA) kits (Sigma–Aldrich, Saint Louis, MO). According to the manufacturer's instruction, the absorbance (A) was detected at 450 nm (A450) and the standard curve was plotted based on the A value.

### 2.10 Immunofluorescence

Immunofluorescence staining was also performed to measure the expression level and detect the location of P2X3 as described previously.^31^ In brief, the rats were trans‐cardially perfused with 100 mL of saline (pH: 7.4) followed by 200 mL of 4% paraformaldehyde in 0.1 M phosphate buffer (pH: 7.4) after deep anaesthetization with 5% chloral hydras and 3% sevoflurane. Then, the lumbar enlargement segments were removed immediately and embedded in an optimal cutting temperature compound. The samples were frozen and sectioned into 10‐μm slices by a freezing microtome (HM550VP, MICROM, GERMAN). For immunofluorescence staining, the slices were incubated with an anti‐P2X3 antibody (1:500, ABclonal) and several cell markers including neuronal nucleus (NeuN) (1:500, Abcam), glial fibrillary acidic protein (GFAP) (1:500, Abcam) and ionized calcium‐binding adapter molecule 1 (Iba‐1) (1:100, Abcam), were added and incubated at 4°C overnight, and then incubated with an Alexa Fluor 488 and 594‐conjugated secondary antibody (1:500, Invitrogen) for 1 h at 37°C. Images were captured with a fluorescence microscope (EVOS FL Auto, Thermo Fisher Scientific).

### 2.11 Statistical analysis

All data were analysed by SPSS version 25.0 software and are expressed as mean ± standard deviation (SD). The results of the behavioural tests were analysed by a two‐way repeated analysis of variance (ANOVA), followed by Bonferroni post hoc comparisons, and the results of western blot, immunofluorescence, ELISA, and quantitative real‐time polymerase chain reaction (qRT–PCR) analyses were tested for significance by one‐way ANOVA with Tukey's post hoc test. *p* < 0.05 was accepted as statistically significant.

## 3 RESULTS

### 3.1 Intrathecal administration of hBMSCs mitigated the mechanical, thermal, and cold hyperalgesia caused by CFA

The total isolated cells revealed high expression of protein markers CD105, CD29, CD73 and CD44 and the absence of CD34, CD45 and CD11b, which was consisted with the surface markers of hBMSCs. Besides, the isolated hBMSCs showed the capacity to differentiate into osteoblasts, adipocytes, and chondrocytes (Data S1). The behavioural test demonstrated that injecting CFA into the plantar surface of the left hind paws of rats created a persistent inflammatory pain model (Figure 1B). The mechanical, thermal, and cold withdrawal thresholds were all significantly reduced after intrathecally administrating hBMSCs for 3 days (*p* < 0.05, Figure 1B–D). As the proinflammatory cytokines are mainly involved in the progression of inflammation, performing with qRT–PCR, western blot, and ELISA, we measured the expression of IL‐1β, IL‐6, and TNF‐α in the spinal cord of the rats. All the results showed that the levels of the proinflammatory cytokine were significantly increased in the CFA‐treated rats, compared with the sham group rats (*p* < 0.05, Figure 1E–G). This founding demonstrated that a successful inflammatory pain model had been established. The data showing that the levels of proinflammatory cytokines were reduced after intrathecal administration of hBMSCs to the CFA‐treated rats obtained via semi‐quantitative analysis, compared with the CFA and CFA+Veh‐1 group (*p* < 0.05, Figure 1E–G). The founding indicated that the inflammation in the CFA‐treated rats had been alleviated by hBMSCs treatment.

### 3.2 Intrathecal administration hBMSCs decreased the expression of P2X3 in the spinal cord of the CFA‐treated rats

**FIGURE 2:**
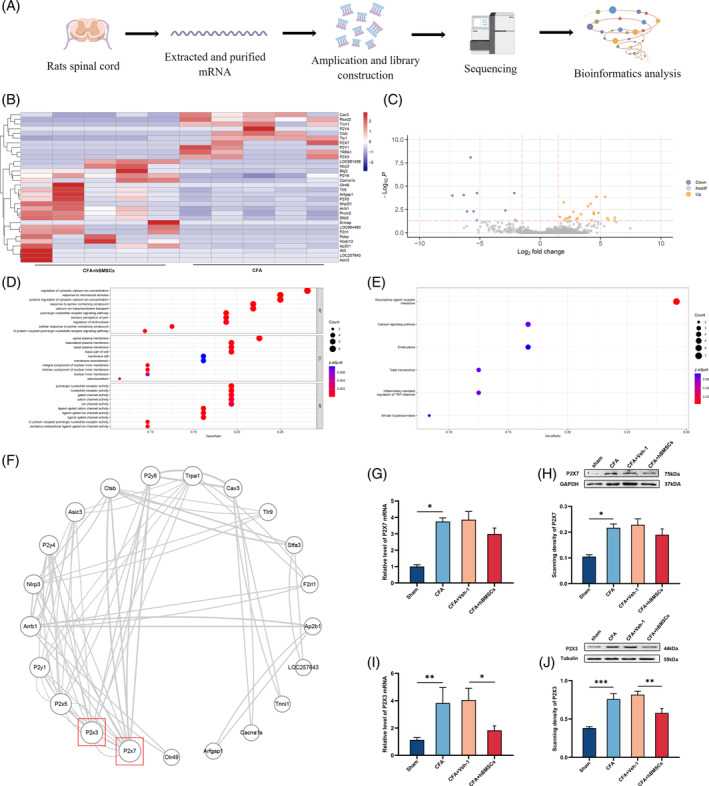
The data of bioinformatics analysis and molecular biology experiment demonstrated that hBMSCs downregulated the expression of P2X3 in CFA‐rats. (A) Schematic overview of high‐throughput sequencing analysis workflow. (B) Heat map of the hierarchical clustering of 32 DEGs between CFA and CFA+ hBMSCs groups. (C) The volcano plot showed the upregulated DEGs (yellow) and downregulated DEGs (blue). (D) GO enrichment analysis revealed the different expressive proteins in BP, CC, and MF. (E) KEGG analysis predicted the probable pathway of DEGs. (F) The PPI network screened the two hub genes in DEGs. (G) The qRT–PCR result of P2X7. (H) Representative western blot image of P2X7 showed that it was no significant difference between CFA and CFA+ hBMSCs groups. GAPDH was the loading control. (I) The qRT–PCR result of P2X3 showed it was significantly decreased in the CFA+hBMSCs group. (J) Representative western blot image of P2X3. Tubulin was the loading control. *p < 0.05, **p < 0.01, ***p < 0.001. *n* = 3 each group.

High‐throughput sequencing was used to screen out the DEGs before/after injecting hMSCs in CFA rats. As shown in Table 2, a total of screen 32 DEGs were screened out. The heat map showed the hierarchical clustering of 32 DEGs expression patterns in CFA and CFA+ hBMSCs groups, and the up‐and down‐regulated genes are coloured in red and blue, respectively (Figure 2A). The Volcano plots demonstrated the 22 up‐regulated and 10 down‐regulated genes, and they were coloured in yellow and blue (Figure 2B). The GO enrichment analysis of the DEGs demonstrated that DEGs were mainly involved in the Regulation of cytosolic calcium ion concentration, Response to mechanical stimulus, Apical plasma membrane, and Purinergic nucleotide receptor activity in BP, CC, and MF (Figure 2C). These results showed the function of mediating the calcium ion concentration and receptor activity of hBMSCs in treating inflammatory pain. The KEGG pathway enrichment analysis showed that the majority of the DEGs were enriched in the Neuroactive ligand‐receptor interaction, Calcium signalling pathway, and Endocytosis (Figure 2D). In addition, as shown in Figure 2E, there were two hub genes in the PPI network, namely P2X3 and P2X7 (Degree = 7 for each protein). The results of qRT‐PCR and western blot showed that the levels of P2X3 and P2X7 in the CFA group were significantly higher than those in the sham group (*p* < 0.01, Figure 2F–I). Intriguingly, only the P2X3 levels were noticeably reduced after hBMSCs treatment (*p* < 0.05, Figure 2F–I). we found the levels of P2X7 were no significant differences between CFA and CFA+ hBMSCs groups.

**TABLE 2:** The detailed information of the 32 upregulated and down‐regulated genes.

Symbol	LogFC	Adjust *p*‐value	Description	Regulation direction (CFA+hBMSCs vs CFA groups)
Rsad2	−7.2797	0.000102	Radical S‐adenosyl methionine domain containing 2	Down
TRPA1	−6.338	9.04E‐05	Transient receptor potential cation channel subfamily A member 1	Down
P2X7	−6.0577	0.005189	Purinergic receptor P2X7	Down
Tnni1	−5.7693	8.18E‐09	Troponin I1, slow skeletal type	Down
P2Y1	−5.5162	0.005123	Pyrimidinergic receptor P2Y1	Down
P2Y4	−5.243	0.04773	Pyrimidinergic receptor P2Y4	Down
Ctsb	−5.2402	5.54E‐05	Cathepsin B	Down
P2X3	−2.8829	0.004049	purinergic receptor P2X 3	Down
Tlx1	−2.1473	5.54E‐05	T‐cell leukaemia homeobox 1	Down
Cav3	−2.0654	0.046398	Caveolin 3	Down
Tlr9	1.6452	0.020319	Toll‐like receptor 9	Up
Arfgap1	1.9414	0.000536	ADP‐ribosylation factor GTPase activating protein 1	Up
P2Y6	2.01635	0.044995	Pyrimidinergic receptor P2Y6	Down
Prom2	2.16345	0.028284	Prominin 2	Up
Btg2	2.36429	0.027135	BTG anti‐proliferation factor 2	Down
Stfa3	2.46522	0.020319	Stefin A3	Up
Arrb1	2.75378	0.005686	Arrestin beta 1	Up
P2X5	3.00281	0.036527	Purinergic receptor P2X 5	Up
Ap2b1	3.11256	0.027135	Adaptor related protein complex 2 subunit beta 1	Up
Cacna1s	3.37802	0.01051	Calcium voltage‐gated channel subunit alpha1 S	Down
LOC684480	3.96863	0.001448	Butyrophilin‐like 9	Up
LOC681458	4.18498	0.000781	Similar to stearoyl‐coenzyme A desaturase 3	Up
Ifit3	4.25848	0.047404	Interferon‐induced protein with tetratricopeptide repeats 3	Down
Nlrp3	4.43659	0.006963	NLR family, pyrin domain containing 3	Up
Ppbp	4.45039	0.009705	Pro‐platelet basic protein	Up
F2rl1	4.65972	0.000139	F2R like trypsin receptor 1	Up
Nhp2l1	4.83183	0.007456	NHP2‐like protein 1	Up
Hoxb13	5.38519	0.000139	Homeo box B13	Up
Ermap	5.57353	0.027146	Erythroblast membrane associated protein	Down
LOC257643	5.68365	0.044995	Cystatin SC	Down
Olr49	6.16416	0.049866	Olfactory receptor family 51 subfamily S member 1	Down
Asic3	6.18452	0.024279	Acid sensing ion channel subunit 3	Up

### 3.3 Cellular co‐localization of P2X3 in the spinal cord

**FIGURE 3:**
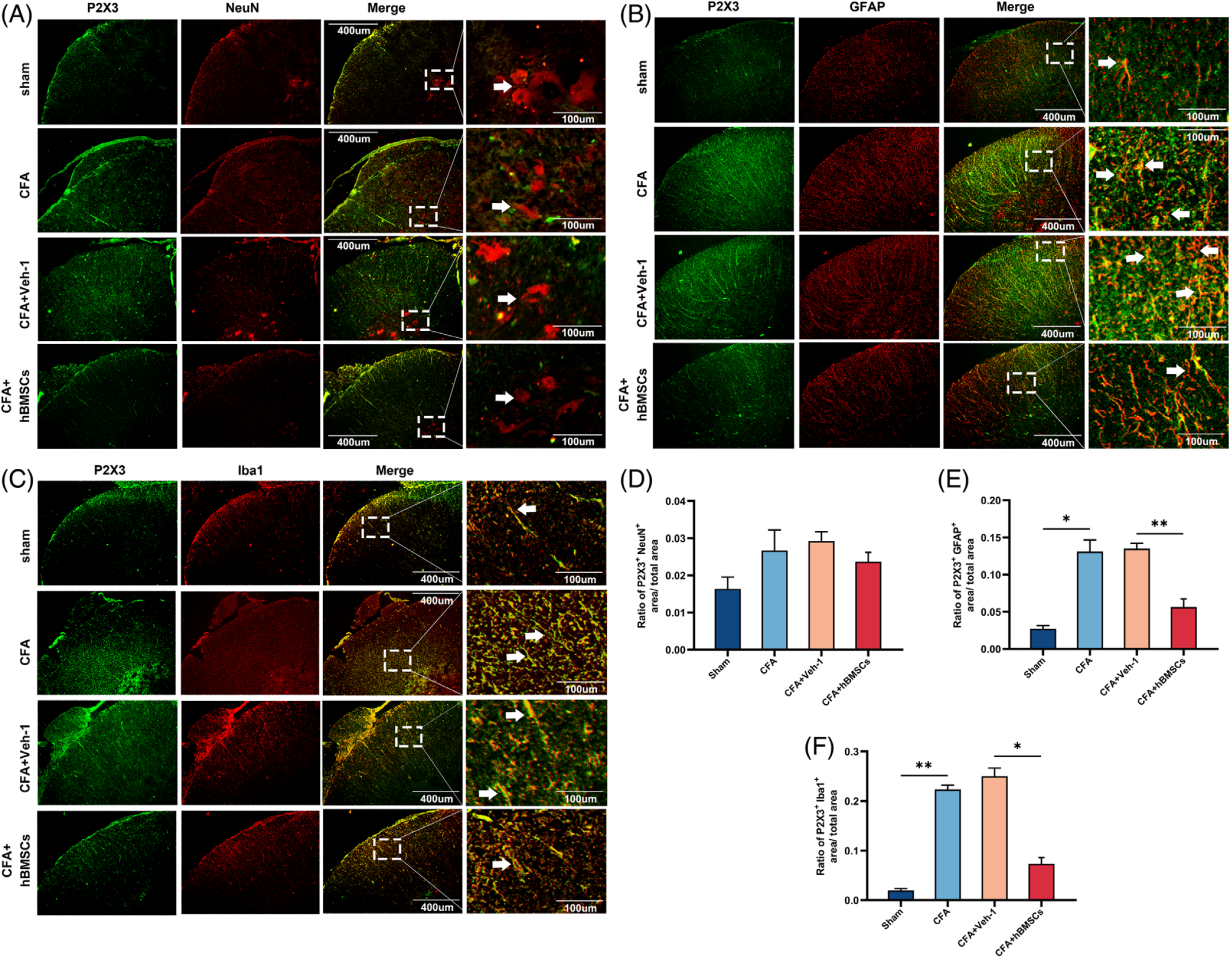
hBMSCs downregulated the expression of P2X3 in nonneural cells in the spinal cord. (A–C) Representative images showed immunofluorescence in the spinal cord indicating that P2X3 colocalized with non‐neural cells in the spinal cord. (D–F) The semi‐quantitative analysis of immunofluorescence images showed that the activity of P2X3 was weakened in the CFA+hBMSCs group, compared with that in the CFA and CFA+Veh‐1 groups. Scale bar = 400 and 100 μm. The white arrows pointed to the spinal cells stained with distinct cell markers. *p < 0.05, **p < 0.01. *n* = 3 each group.

To identify which cells exerted the main affected on the expression of P2X3 in the spinal cord tissue, we used three cell markers, NeuN, GFAP, and Iba1 for double‐staining with P2X3. The immunofluorescence images of double‐stained cells demonstrated that the levels of P2X3 were increased and that the protein was mainly localized to GFAP‐marked astrocytes and Iba1‐ marked microglia, not neurons in the spinal cords (Figure 3A, B, C). According to the semi‐quantitative analysis of these immunofluorescence images, no significant differences in P2X3 colocalization with NeuN were found in any group, but the P2X3 colocalization with GFAP and Iba1 was increased in the spinal cord of the rats of the CFA and CFA+Veh‐1 groups, compared with the sham group, and the expression of these markers was downregulated in the CFA+hBMSCs group (*p* < 0.05, Figure 3D–F). These results showed that P2X3 was mainly expressed in glial cells during inflammation that caused pain.

### 3.4 The levels of P2X3 in the spinal cord are associated with persistent inflammatory pain

**FIGURE 4:**
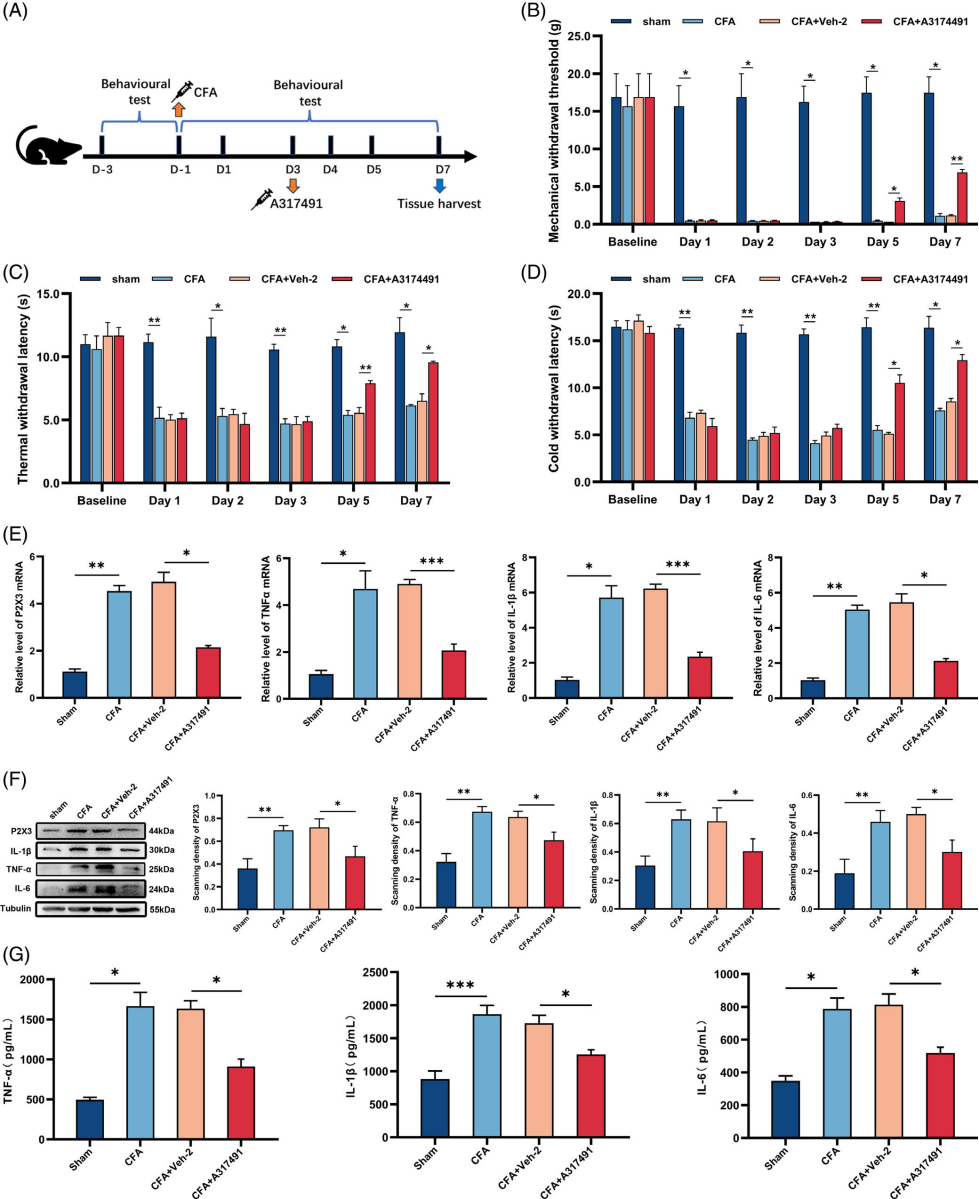
A317491 inhibited the upregulation of P2X3 and inflammatory cytokines in the spinal cord. (A) The procedure of intrathecally administrating A317491. (B–D) The analgesic effect of intrathecal injection A317491 on the mechanical, thermal, and cold withdrawal thresholds. The CFA+A317491 group exhibited a significant increase in mechanical, thermal, and cold withdrawal thresholds compared with those in the CFA and CFA+Veh‐2 groups. (E) The qRT–PCR result for the gene expression of P2X3 and inflammatory cytokines. The mRNA levels of P2X3, IL‐1β, TNF‐α, and IL‐6 were increased in the CFA and CFA+ Veh‐2 groups, while they were decreased in CFA+A317491 group. (F) Representative western blot image showed each protein and the quantification of their relative expression in each group. Tubulin was the loading control. The changes in the levels of each protein were the same as qRT‐PCR. (G) The levels of inflammatory cytokines were determined by ELISAs. The changes in the levels of inflammatory cytokines were the same as those obtained by western blot analysis. *p < 0.05, **p < 0.01, ***p < 0.001. *n* = 3 each group.

**FIGURE 5:**
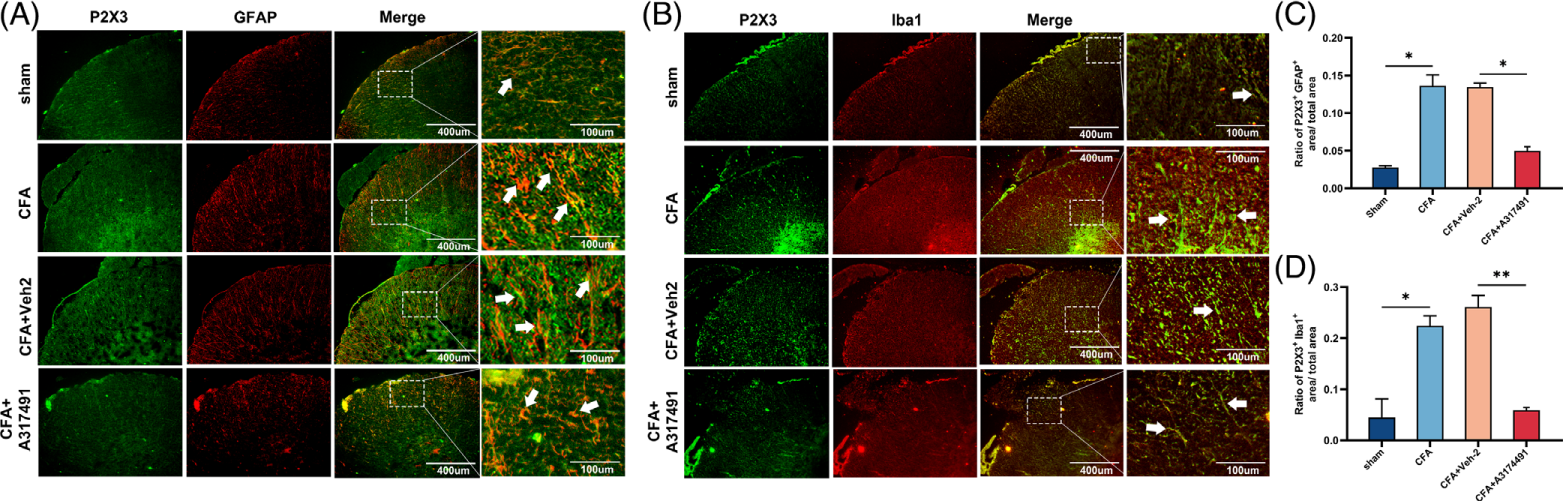
A317491 inhibited the activity of P2X3 in the spinal cord. (A, B) Representative images of immunofluorescence staining for P2X3 colocalized with astrocytes and microglia in each group. (C, D) The semi‐quantitative analysis of immunofluorescence images showed that intrathecally administrated A317491 downregulated P2X3 expression in the spinal cord in CFA‐treated rats, compared with the CFA and CFA+Veh‐2 groups. Scale bar = 400 and 100 μm. The white arrows pointed to the spinal cells stained with distinct cell markers. *p < 0.05, **p < 0.01. *n* = 3 each group.

In this study, according to the results of behavioural tests and qRT–PCR, western blot, and ELISA of P2X3, we found that when the rats were in hyperalgesia after CFA injection, the levels of P2X3 increased obviously. When we observed attenuation in hypersensitivity in CFA rats treated with hBMSCs, the levels of P2X3 were also found to be downregulated in the spinal cord of the CFA rats. Therefore, a P2X3 inhibitor group, CFA+A317491, was added to the present study to explore the role of P2X3 in this process. The procedure of this experiment is shown in Figure 4A. We found that the analgesic effect of injecting the P2X3 antagonist was similar to that of intrathecally administrating hBMSCs, compared with that of the CFA group, according to the behavioural tests (*p* < 0.05, Figure 4B–D). The results of the qRT–PCR, western blot, and ELISA of P2X3 and proinflammatory cytokines were also downregulated after the intrathecal administration of hBMSCs in the CFA+A317491 group, compared with the CFA and CFA+Veh‐2 groups (*p* < 0.05, Figure 4E–G). The representative pictures and the semi‐quantitative analysis of immunofluorescence also showed a decrease in the expression of the P2X3 in the CFA+A317491 group, compared with the CFA and CFA+Veh‐2 groups (*p* < 0.05, Figure 5A–D). These results illustrated that hBMSCs alleviated inflammatory pain mainly by inhibiting the expression of P2X3 and that the levels of P2X3 could affect the production of proinflammatory cytokines.

### 3.5 Upregulating the expression of P2X3 reversed the analgesic effects of intrathecally administrated hBMSCs

**FIGURE 6:**
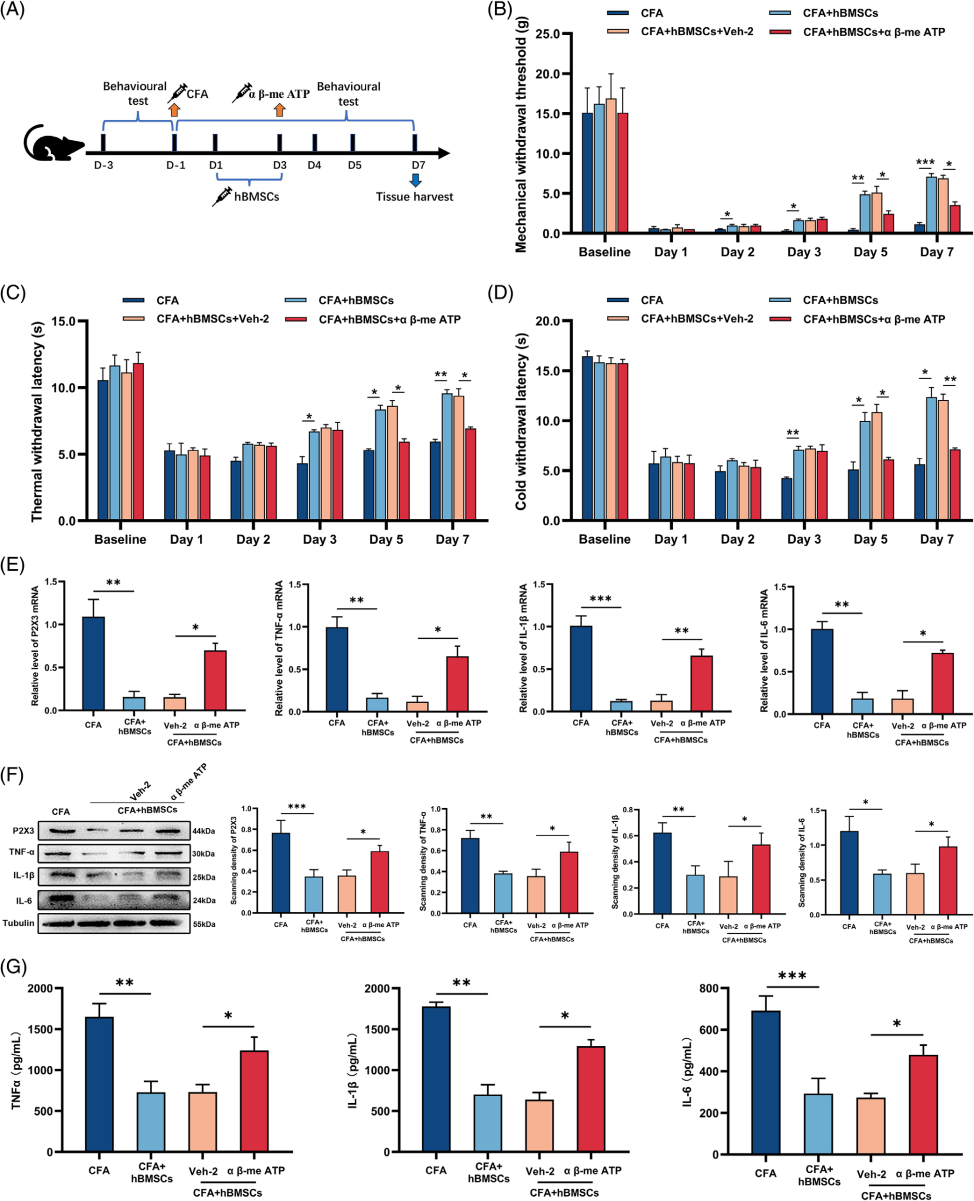
α β‐me ATP decreased the analgesic effect of intrathecally administrated hBMSCs. (A) The procedure of intrathecally administrating α β‐me ATP experiment. (B–D) Behavioural tests demonstrated that applying α β‐me ATP decreased the analgesic effect of the hBMSCs. (E) The qRT–PCR result for the gene expression of P2X3 and inflammatory cytokines demonstrated that intrathecally injecting α β‐me ATP reduced the analgesic effect induced by hBMSCs. (F) Representative image and the quantification of the relative expression of each protein in the spinal cords of rats. Tubulin was the loading control. The changes in the levels of each protein were the same as those obtained by qRT–PCR. (G) The result of each protein was determined by ELISA. The changes in the levels of inflammatory cytokines were the same as they were in the western blot analysis. *p < 0.05, **p < 0.01, ***p < 0.001. *n* = 3 each group.

**FIGURE 7:**
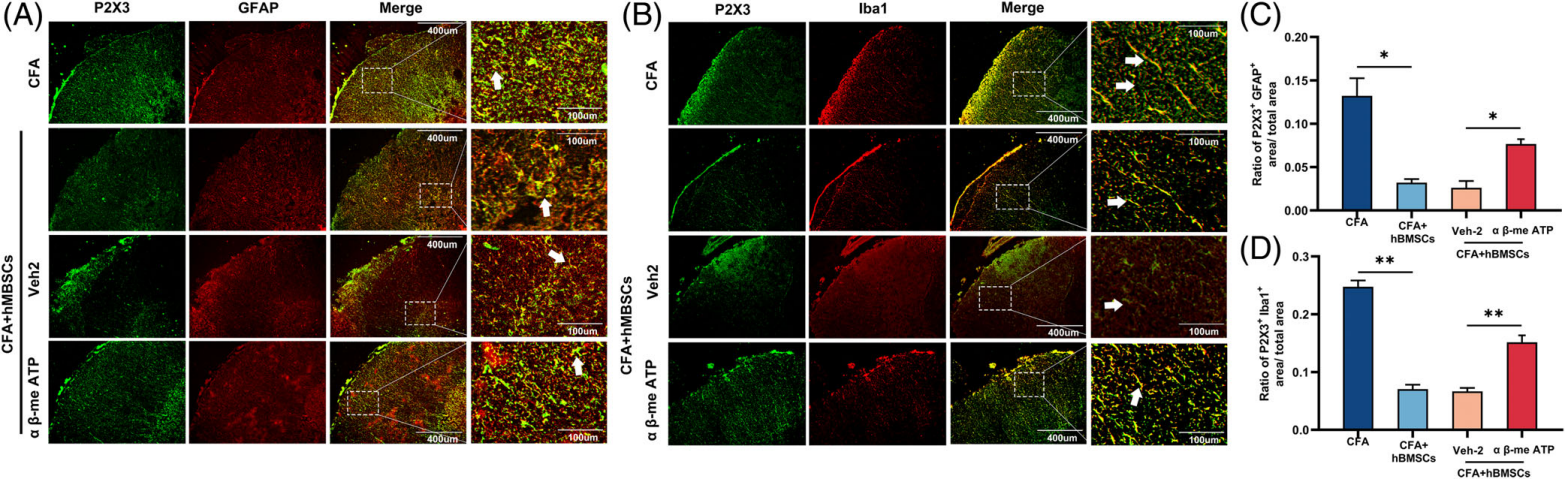
α β‐me ATP increased the activity of P2X3 in CFA rats intrathecally administrated hBMSCs. (A,B) Representative immunofluorescence images showed P2X3 colocalized with astrocytes and microglia. (C,D) The semi‐quantitative analysis of the immunofluorescence images demonstrated that intrathecal injection of α β‐me ATP attenuated the decrease in P2X3 expression followed by intrathecal administration of hBMSCs in the spinal cord of CFA‐treated rats. The white arrows indicate P2X3 co‐staining in distinct cells. Scale bar = 400 and 100 μm. *p < 0.05, **p < 0.01, ***p < 0.001. *n* = 3 each group.

After injecting the antagonist of P2X3, we found that its analgesic effect in treating inflammatory pain was similar to that of hBMSCs. To investigate whether hBMSCs made an alleviation mainly by downregulating P2X3, we injected the agonist of P2X3, α β‐me ATP, after administrating hBMSCs, and the procedure of the experiment is shown in Figure 6A. The behavioural tests showed that α β‐me ATP could reverse the analgesic effect brought by hBMSCs, compared with the CFA+hBMSCs group (*p* < 0.05, Figure 6B–D). The results of qRT–PCR, western blot, and ELISA of the P2X3 and proinflammatory cytokines also showed an increase in the CFA+hBMSCs+α β‐me ATP group, compared with the CFA+hBMSCs group (*p* < 0.05, Figure 6E–G). The expression of P2X3 was also upregulated in the representative pictures of immunofluorescence in the spinal cord of the rats according to the semi‐quantitative analysis (*p* < 0.05, Figure 7A–D). These results supported that hBMSCs alleviated inflammatory pain mainly by inhibiting the expression of P2X3.

## 4 DISCUSSION

Accumulating evidence has indicated that the injection of CFA into the rodent hind paw could lead to sustained inflammation characterized by persistent hypersensitivities.^19^, ^20^ For example, nerve growth factor (NGF) is considered a crucial factor in inflammation and hyperalgesia, and its level was elevated in the paws of inflamed rats treated with CFA.^34^ In addition, elevated NGF expression was also detected in rodent DRG after administration of CFA, which induces sensitization of transient potential receptor V1 (TRPV1) and/or A1 (TRPA1)‐positive DRG sensory neurons and in turn facilitates the transmission of pain signals to the central nervous system.^35^ Thus, the CFA‐induced chronic pain rat model is commonly regarded as an inflammatory pain model. Based on this CFA‐injection rodent model, we found that intrathecal administration of hBMSCs could mitigate the CFA‐induced mechanical, thermal, and cold hyperalgesia by downregulating the expression of P2X3 in spinal astrocytes and microglia. Moreover, the levels of proinflammatory cytokines, including IL‐1β, IL‐6, and TNF‐α, were also decreased after treatment with hBMSCs.

Inflammatory pain is a complex process which had not been clearly elucidated the potential mechanisms now. Our high‐throughput sequencing result screened out that there were 32 DEGs between CFA and CFA+ hBMSCs groups, and the GO term enrichments showed that the 32 DEGs were significantly enriched in the Regulation of cytosolic calcium ion concentration in BP, Apical plasma membrane in CC, and Purinergic nucleotide activity in MF. Accumulating evidence has indicated that inflammatory pain can behave as a dysfunction of the calcium ion concentration and the activation of purine receptors.^36^, ^37^ This is consistent with the results of our GO analysis. In the KEGG enrichment analysis, we found that Neuroactive ligand‐receptor interaction was the most significantly enriched, and the Calcium signalling pathway was the secondary significantly enriched. These demonstrated that calcium may play a vital role in transmitting the signal of the stimuli to inflammatory pain, and this founding had been proven in a previous study.^38^ In the PPI network analysis, P2X3 and P2X7 were identified as the hub genes. Numerous reports have proven that the purinergic receptor P2X3 plays a pivotal role in transmitting stimuli signals in inflammatory pain, and allodynia can be alleviated by inhibiting P2X3.^39^, ^40^, ^41^ In addition, P2X7 as the other purinergic receptors, studies had also been performed to confirm its importance in the development of inflammatory pain.^42^, ^43^ In our study, we found that the levels of P2X3 and P2X7 increased in the spinal cord after injecting CFA into the plantar surface of the left hind paws of the rats, which was the same as previous studies mentioned.^23^, ^24^ Interestingly, only the inhibition of P2X3 was found in the CFA rats treated with hBMSCs, while no significant differences were observed in the expression of P2X7 before/after injecting hBMSCs. In other words, hBMSCs attenuate CAF‐induced inflammatory pain by inhibiting the expression of p2X3. In sum, we were the first time to use hBMSCs to treat CFA‐ induced inflammatory pain in rats, and the results showed that the expression of P2X3 in the spinal cord decreased following the attenuation of CFA‐induced hypersensitivity.

Clinical and animal studies have reported that the intrathecal administration of MSCs have a significant effect on pain.^15^ Chen et al. found that the survival of transplanted BMSCs could last for 84 days after intrathecal injection,^44^ and a recent systematic review had indicated that the migration of transplanted BMSCs after intrathecal injection was directed towards the surface of dorsal spinal cord on the ipsilateral peripheral nerve injury side.^45^ These findings ensured the efficacy and safety of BMSCs intrathecal transplantation. Moreover, it was indicated that MSCs could repress the inflammatory reaction and attenuate hypersensitivity in rats under chronic pain conditions.^46^, ^47^ In the present study, the concentrations of IL‐1β, IL‐6, and TNF‐α were all decreased by determination of western blot, qRT–PCR, and ELISA after administration of hBMSCs, which conformed to the early report that MSCs could reduce the secretion of proinflammatory cytokines.

Studies have illustrated that injecting MSCs into rats with neuropathic pain can inhibit the activation of microglia and astrocytes in the spinal cord.^48^ To identify the cells that mainly influence the levels of P2X3 in the spinal cord, we used P2X3 to double‐staining with NeuN, GFAP, and Iba1. Interestingly, we found that microglia and astrocytes, but not neurons, were colocalized with P2X3. Whitehead et al. reported that glial cells were the main sources of intrathecal TNF‐a, IL‐1β, and IL‐6 in allodynia.^49^ Therefore, we speculated that neurons did not participate in the development of inflammatory pain. Moreover, we found that the changes in the levels of proinflammatory cytokines followed the changes in P2X3, and Teixeira et al. demonstrated that inhibiting P2X3 expression reduced the levels of proinflammatory cytokines, including TNF‐α and IL‐6.^50^ Xia et al. proved that knocking out P2X3 suppressed the activation of microglia and alleviated neuropathic pain.^51^ Taken together, these studies suggested that P2X3 regulated the activation and reproduction of glial cells via an uncertain mechanism, and indirectly induced the glial cells to secrete the proinflammatory cytokines. Thus, considering our results and these studies, we hypothesized that MSCs attenuated CFA‐induced hyperalgesia mainly by hindering the expression of P2X3.

To verify that P2X3 plays a critical role in the pathological process of inflammatory pain, an antagonist of P2X3, A317491, or an agonist of P2X3, α β‐me ATP, was injected into CFA rats. A317491, a selective antagonist of P2X3, is regarded as an effective medicine to attenuate inflammatory pain.^52^ In the present study, the behavioural tests showed that treatment with A317491 markedly reversed hypersensitivity in CFA‐treated rats. Similarly, the molecular and cellular changes also revealed that the levels of P2X3 and the proinflammatory cytokines were reduced in the CFA+ A317491 group. Namely, the analgesic effect of injecting A317491 is the same as that of injecting hBMSCs. However, the administration of A317491 did not restore the health of the CFA‐treated rats. The reason for this failure to restore may involve other unidentified factors, such as NLRP and CB2, that contribute to the CFA‐induced inflammatory pain.^53^, ^54^ After the CFA‐treated rats were injected α β‐me ATP, the behavioural tests showed a reduction in the analgesic effect of administrating hBMSCs, and the results of immunohistochemical staining showed the expression of P2X3 in the spinal cord was increased compared with that in the CFA+hBMSCs group. It was suggested that the increased expression of P2X3 receptors might potentiate the production of inflammatory mediators.^55^ Moreover, a recent study reported that the blockage of P2X3 decreased the activation of NLRP3 inflammasome and subsequent cleavage of caspase‐1 which modulated the levels of some inflammatory mediators, such as IL‐1β and IL‐18.^56^ These findings demonstrated the critical role of P2X3 receptor on mediating the activation of inflammatory mediators, and led us to hypothesize that the BMSCs could suppress the inflammatory mediators through specifically targeting the activation of P2X3 receptor.

There are some limitations to the current study. Firstly, a recent review had indicated the BMSCs might play important role in the activity of osteoclasts and osteoblasts.^57^ However, as the main purpose of this study was to assess the therapeutic effect of BMSCs against inflammatory painful behaviours, we did not provide experimental evidence for the effects of BMSCs on osteoclast formation and bone resorption in bone tissues, which was in accordance to other studies that focused on the effect of BMSCs intrathecal injection on chronic pain.^45^, ^58^ More researches on the effects of BMSCs on the activities of osteoclasts and osteoblasts are urgently needed in the future. In addition, we measured only the effects of intrathecal administration of BMSCs on the levels of P2X3 and the proinflammatory cytokines in the spinal cord. The influence of BMSCs administrated through other routes and other tissues needs to be determined through further research.

## 5 CONCLUSION

In summary, we demonstrated that hBMSCs relieved hypersensitivity to CFA‐induced inflammatory pain by inhibiting the expression of P2X3, which suggested a new therapy for treating inflammatory pain. Furthermore, we found that P2X3 played a vital role in signalling inflammatory pain pathways and that P2X3 may be a new target for treating inflammatory pain.

## AUTHOR CONTRIBUTIONS

Xiang Li and Dongdong Yuan were responsible for the conception, study design, and final editorial decision of the manuscript. Zhizhao Deng was responsible for the study design. Yifan Deng and Zhizhao Deng were responsible for the study design, data collection and analysis, and manuscript writing and revision. Jianfen Liang contributed to the experimental studies and data collection. Zhenye Zhang was responsible for the group allocation. The final version of the manuscript was read and approved by all the authors.

## FUNDING INFORMATION

This study was supported by The Basic and Applied Basic Research Foundation of Guangdong Province (2021A1515220081); The Science and Technology Program of Guangzhou (202201020446); The Fundamental Research Funds for the Central Universities, Sun Yat‐sen University (22qntd3401); National Natural Science Foundation of China (82072216 and 81871597); and the Guangdong Basic and Applied Basic Research Foundation (2019A1515010093).

## CONFLICT OF INTEREST STATEMENT

The authors declare that they have no competing interests.

## Supporting information


**Data S1.** Supporting Information.

## Data Availability

The data and materials in the current study are available from the corresponding author on reasonable request.
