# Supplementary material for: Bone marrow‐derived mesenchymal stem cells attenuate complete Freund's adjuvant‐induced inflammatory pain by inhibiting the expression of P2X3
Source: Cell Prolif. 2023 Mar 27;56(10):e13461. doi: 10.1111/cpr.13461 (PMC10542618; doi:10.1111/cpr.13461)
Supplement: Supplementary file 1 — Data S1. Supporting Information. [file CPR-56-e13461-s001.pdf]

OriCell®

产品检测报告

OriCell®成人骨髓间充质干细胞

产品货号：HUXMA-01001

We help you discover life

OriCell®

基本信息

产品名称	**OriCell®成人骨髓间充质干细胞**
货号	**HUXMA-01001**
批号	211102H61
供体信息	男
冻存代次	**P2**
保存条件	液氮（-196℃）

检测标准及结果

检测项目			检测结果	检测标准	结论
常规检测	细菌、真菌		阴性	阴性	合格
	支原体		阴性	阴性	合格
	内毒素		≤10EU	≤10EU	合格
鉴定检测	复苏存活率		98.07%	≥80%	合格
	活细胞数		1.90×10^{6}	$\geq1\times10^{6}$	合格
	细胞复苏贴壁率		99.06%	≥80%	合格
	生长状态		形态呈长梭性，呈极性排列，群体倍增时间为 25.74h	形态为长梭形，呈极性排列；群体倍增时间≤ 72 h	合格
	分化能力		可分化为成脂、成骨、成软骨细胞	经定向诱导，可分化为成骨细胞、脂肪细胞、成软骨细胞	合格
	表面标记分子	**CD105**	99.13%	⩾70%	合格
		**CD29**	82.93%	⩾70%	合格
		**CD73**	99.97%	⩾70%	合格
		**CD34**	0.72%	⩽5%	合格
		**CD45**	0.28%	⩽5%	合格
		**CD11b**	0.39%	⩽5%	合格
		**CD44**	99.68%	⩾70%	合格

细胞生长状态

细胞增殖能力

选取对数期（1-3 天）数据进行计算，得出细胞群体倍增时间为 25.74h。

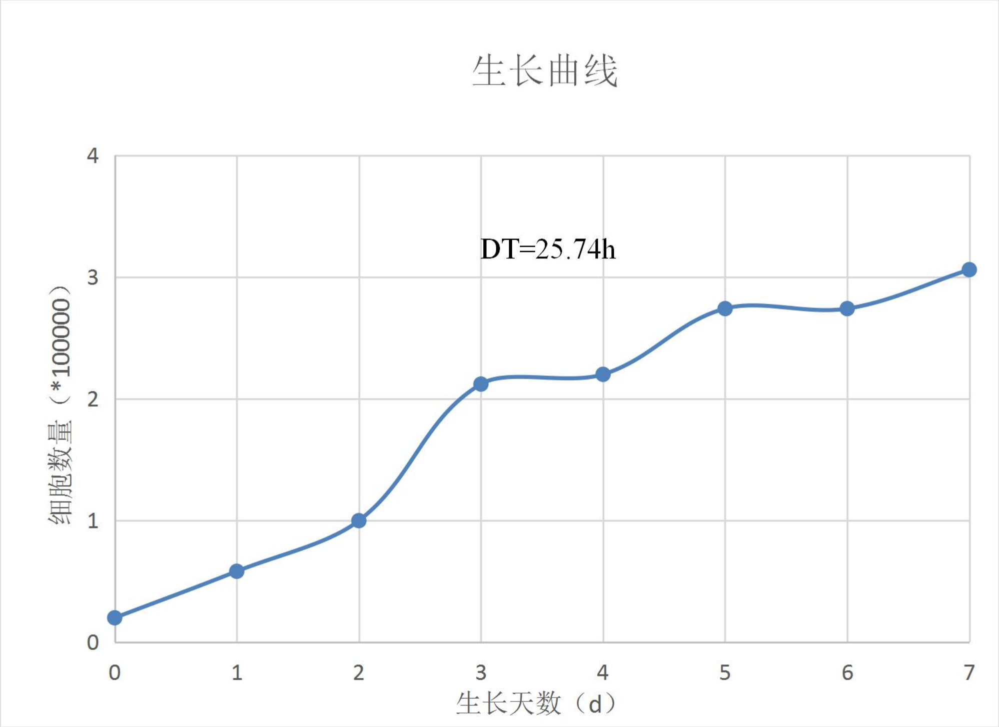

细胞传代能力

细胞均呈长梭形生长，极性良好，立体感良好。经 5 次传代后，细胞仍较有活力。

本批次细胞 Pn+1 代生长 48h 后的形态

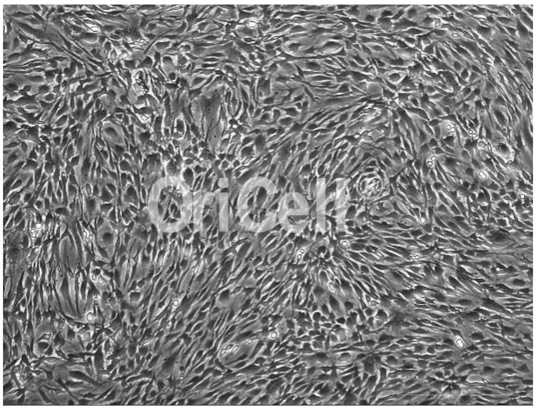

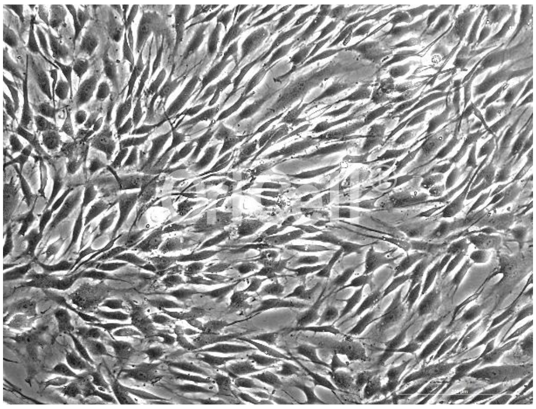

本批次细胞 Pn+3 代生长 48h 后的形态

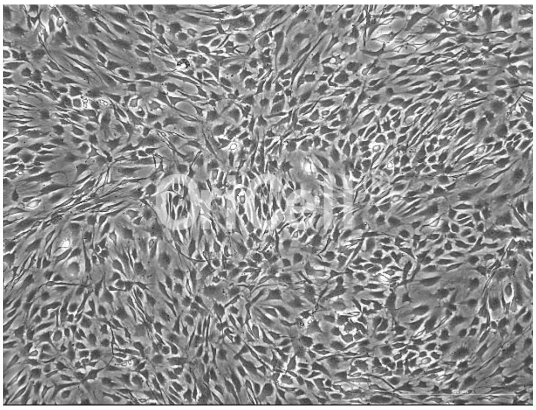

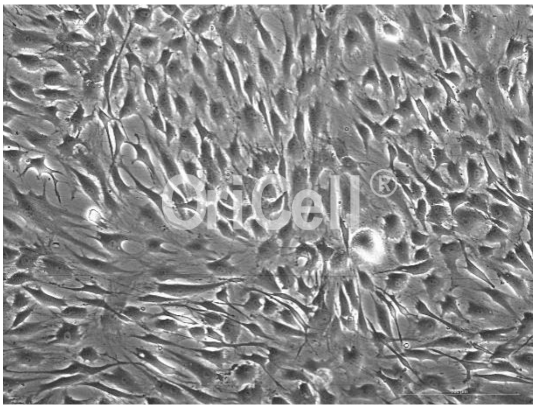

本批次细胞 Pn+5 代生长 48h 后的形态

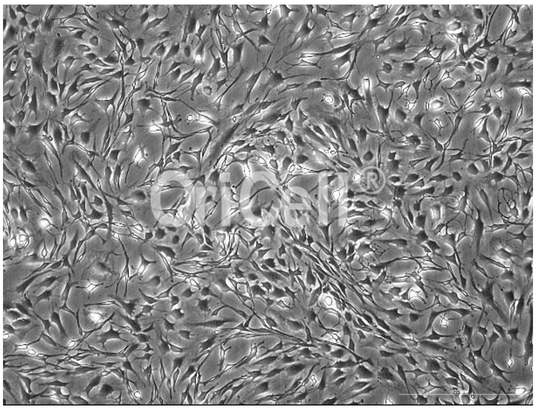

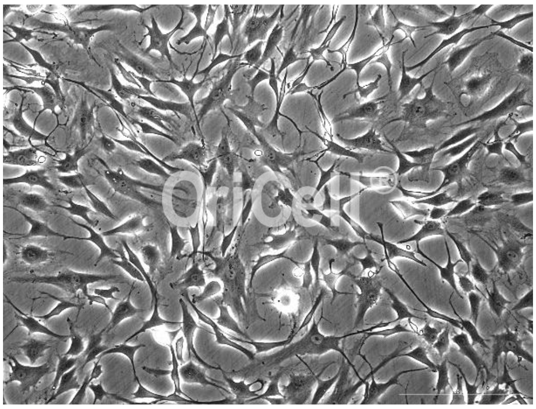

诱导分化能力

成脂诱导分化

细胞汇合度约达 90%时，加入间质干细胞成脂诱导液，25 天后，进行油红 O 染色，可见被染为红色的较标准的脂滴。

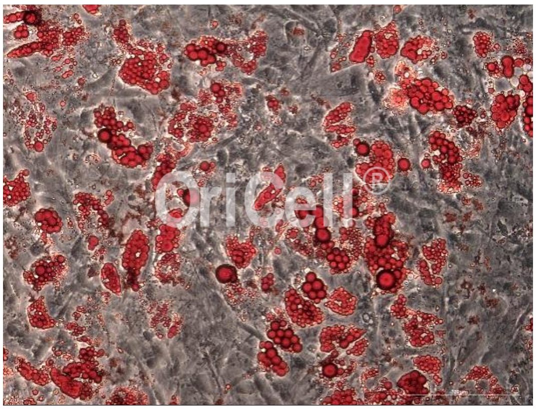
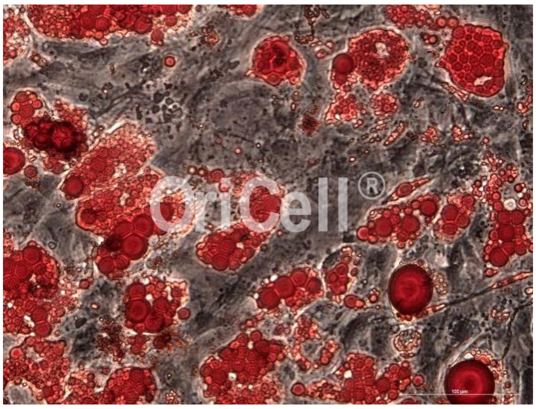

成骨诱导分化

细胞汇合度约达 70%时，加入间质干细胞成骨诱导液，17 天后进行茜素红染色，茜素红与类骨质结合，形成同心圆状的深红色小结节。

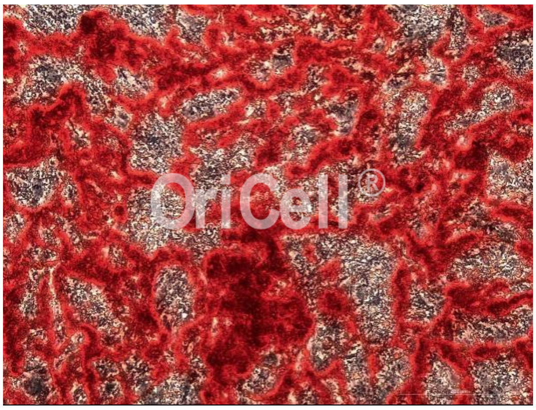
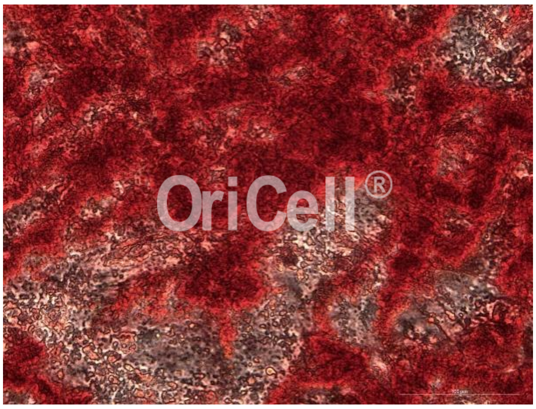

成软骨诱导分化

细胞经团块培养，间质干细胞软骨诱导液诱导培养，细胞从贴附于离心管底部的扁平细胞团逐渐变成细胞球，诱导 21 天后，细胞团变大，变圆，表面变得光滑。

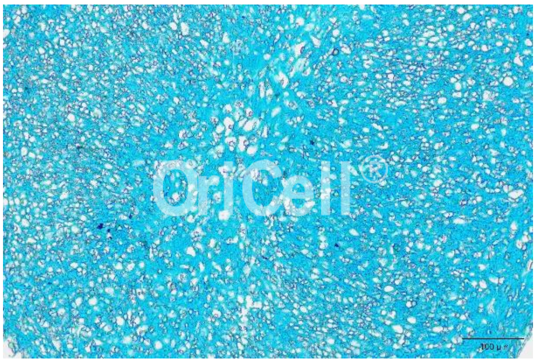

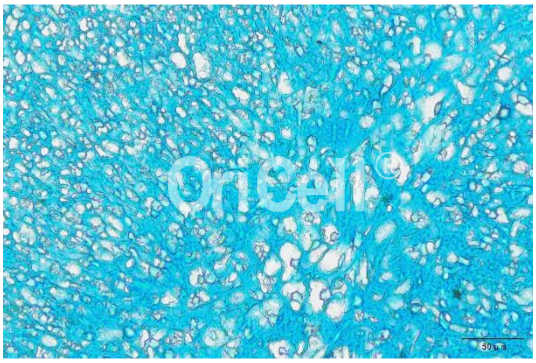

表面分子流式检测

数据详情

Mouse IgG1, κ Isotype Control Antibody

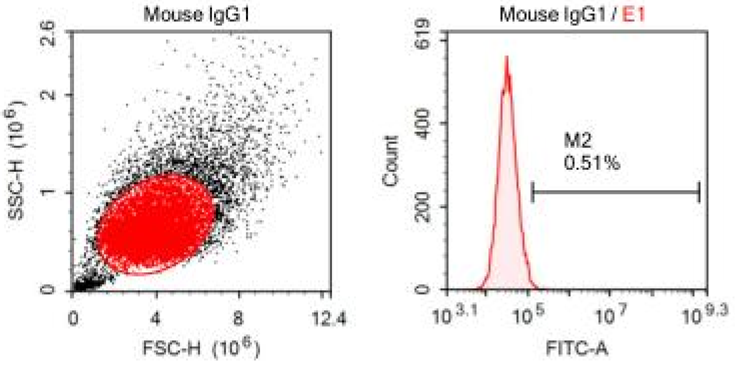

Anti-human CD105 antibody

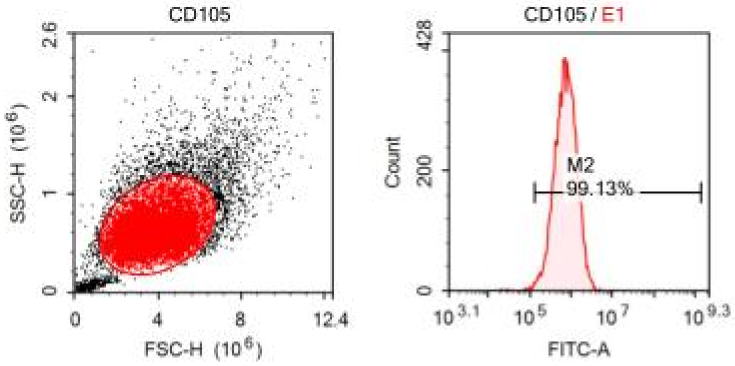

Anti-human CD29 antibody

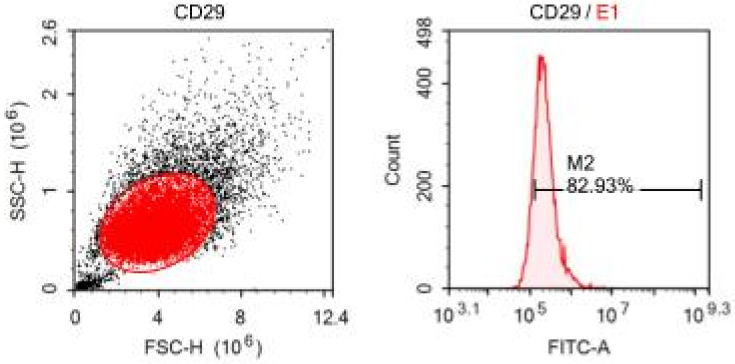

Anti-human CD73 antibody

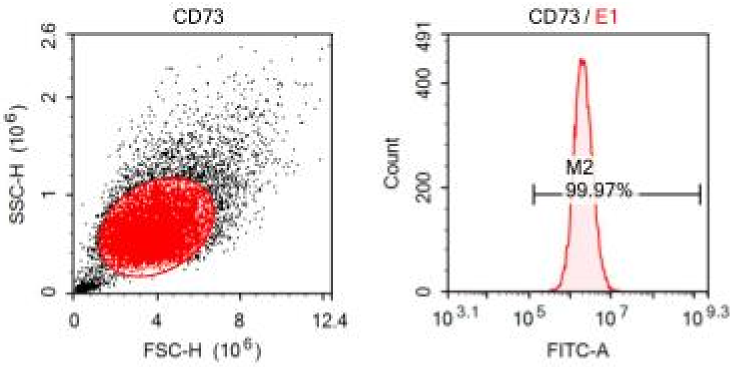

Anti-human CD34 antibody

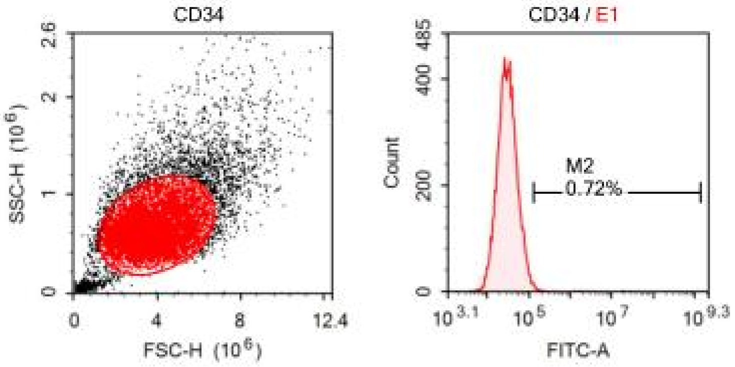

Anti-human CD45 antibody

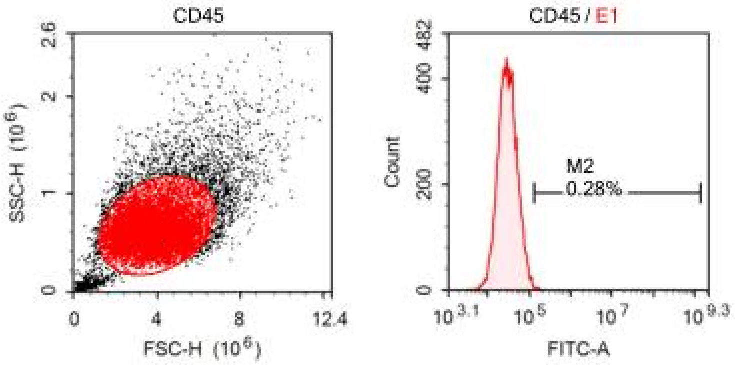

Anti-human CD11b antibody

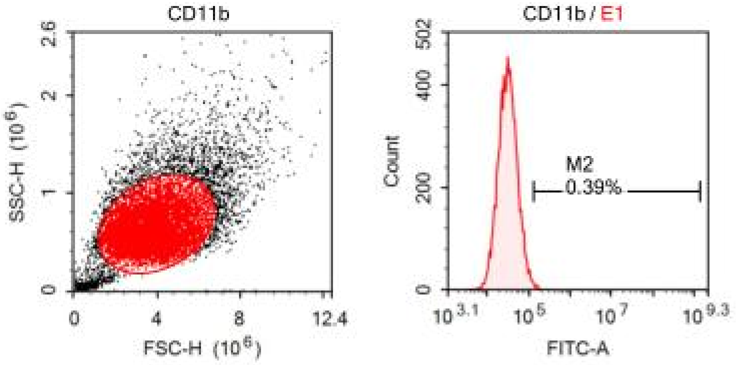

Rat IgG2b, κ Isotype Control Antibody

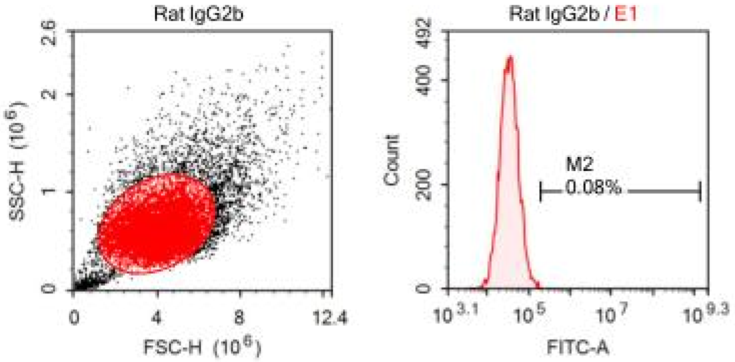

Anti-human CD44 antibody

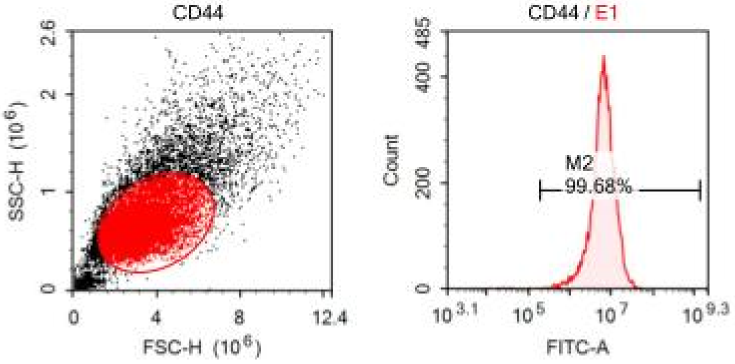

结论

本品经检测符合各项指标要求，准予放行。

检验人员：Nancy

审核人员：Glory

赛业（广州）生物科技有限公司
检验合格

赛业（广州）生物科技有限公司保留OriCell®细胞培养产品技术文件的所有权利。

没有赛业（广州）生物科技有限公司的书面许可，本文件的任何部分，

不得改编或转载用作其他商业用途。
